# Ethnobotanical survey of wild edible plants used by Baka people in southeastern Cameroon

**DOI:** 10.1186/s13002-020-00413-0

**Published:** 2020-10-22

**Authors:** Pascal Eric Billong Fils, Natacha Afiong Nana, Jean Lagarde Betti, Oumar Farick Njimbam, Stéphanie Tientcheu Womeni, Eva Ávila Martin, Guillermo Ros Brull, Robert Okale, Julia E. Fa, Stephan M. Funk

**Affiliations:** 1 Department of Plant Biology, Faculty of Sciences, University of Douala, BP 24 157 Douala, Cameroon; 2 Zerca y Lejos ONGD, c/Sambara 128, 28027 Madrid, Spain; 3 Department of Natural Sciences, School of Science and the Environment, Manchester Metropolitan University, Manchester, M1 5GD UK; 4 Center for International Forestry Research (CIFOR), CIFOR Headquarters, Bogor, 16115 Indonesia; 5 Nature Heritage, St. Lawrence, Jersey, Channel Islands UK

**Keywords:** Dja biosphere reserve, Wild edible plants, Ethnobotany, Diversity indexes, Baka people, Hunter-gatherer, Africa, Food security

## Abstract

**Background:**

Forest inhabitants worldwide, and indigenous people especially, have depended for generations on plants and animals harvested in these ecosystems. A number of Baka hunter-gatherer populations in south-eastern Cameroon became sedentarised in the 1950s, but still rely on hunting and gathering to meet their basic needs. The use of wild edible plants (WEP) by these communities remains largely undocumented. In this study, we record the diversity of WEP used by Baka people in dense rainforests in the Mintom region. The area still contains relatively undisturbed forest expanses, just south of the Dja Biosphere Reserve, one of the most important protected areas in the Congo Basin.

**Methods:**

We conducted two ethnobotanical surveys in 2019 in four villages on the Mintom road. In the first survey, we interviewed a total of 73 individuals to determine WEP usage. In our second survey, we specifically quantified WEP harvested and consumed daily in a number of households over a 2-week period during the major rainy season, when use of forest products is highest. Specimens of all recorded plants were collected and identified at the National Herbarium of Cameroon.

**Results:**

We documented 88 plant species and 119 unique species/plant organ/recipes in 1519 different citations. A total of 61 genera and 43 families were noted. Excluding 14 unidentified wild yam species, 17 WEP species had not been reported in previous ethnobotanical surveys of the Baka. Our results showed that cultivated starchy plant foods make up a significant proportion of our study population’s daily nutritional intake.

**Conclusions:**

A high diversity of WEP is consumed by the studied Baka communities. The study area is likely to be significant in terms of WEP diversity since 18 out of the 30 “key” non-timber forest products, NTFP, in Cameroon were mentioned. Documentation of the use of WEP by indigenous communities is vital to ensure the continuity of traditional knowledge and future food security.

## Background

In tropical forests throughout the globe, wild edible plants (WEP) and fungi have great cultural significance as well as conferring nutritional benefits for myriad indigenous farming and hunter-gatherer communities [1]. These foods provide a variety of macro- and micronutrients across different seasons and ecological zones [2], but can also be important famine foods [3, 4]. Some WEP plays a symbolic link between nature and society for those communities who use them. African hunter-gatherer populations consider yam tubers to have a connection between elephants and their tutelary spirit “jengi,” since wild yams are a fundamental plant food for Pygmies and elephants—elephant hunting is traditional in these indigenous groups [5].

The diet of indigenous peoples in general, and hunter-gatherers in particular, are rapidly changing [6]. Currently, almost no hunter-gatherer population relies solely on wild foods, consuming a mixed diet that includes farmed foods, and in some cases diets that are subsidized by governments and aid organizations [7]. Reyes-García et al. [8] reported that Baka Pygmies living in or near market towns in Cameroon had a lower dietary diversity and consumed more sugar than those living more remotely whose diets contained more WEP and were more balanced in micronutrients. After sedentarization from the 1950s onwards, Baka who supplemented their life in the village with time in forest camps exhibited reduced stress levels helping them maintain a better nutritional status overall [9].

Market economies impact the lifestyle of hunter-gatherers, often by increasing their reliance on cultivated starchy staple foods and decreasing the use of WEP, eroding traditional local knowledge on how to find, identify, and process these plants. For example, only a few Baka elders still mastered the preparation of African oil bean (*Pentaclethra macrophylla*) seeds, which require several days soaking in running water to eliminate toxic compounds [10]. Bahuchet et al. [11] suspected that knowledge of the use of some WEP has already completely disappeared. Gallois et al. [10] documented how the high valuation of cultivated and commercial foods has changed the vocabulary used by the Baka to describe wild foods. The bark of *Afrostyrax lepidophyllus* is now known as “[bouillon] cubes of the forest” to the Baka, reflecting that bouillon cubes are, together with salt, the most bought dietary item by these communities when opportunities arise. In parallel to these social changes, the environment is being degraded at an unprecedented scale. Between 2000 and 2014, 16.6 million hectares of rainforest were lost in the Congo Basin, most (84%) from small-scale, nonmechanized forest clearing for agriculture, alongside selective logging [12].

Few studies have cataloged and investigated the use of WEP among hunter-gatherers in the Congo Basin. Hunter-gatherers are known for their extensive knowledge of plants used for medicine, food, and their material culture; as many as 24 plants (77%) used by BaYaka hunter-gatherers from Congo have bioactive properties and some are positively associated with children’s BMI [13]. Studies on general plant use have been conducted among hunter-gatherers but most focus on medicinal plants rather than WEP. Studies on WEP have concentrated on wild yam tubers because of their importance for understanding the colonization of the rainforest by hunter-gatherer populations [14–18]. Only Tanno [19] for the Mbuti and Gallois et al. [10] and Betti et al. [20–22] for the Baka have investigated the broad spectrum of use of WEP. Hattori [23] detailed the use of Marantaceae plants as non-timber forest products, NTFP, two of which (*Haumania danckelmaniana*, *Trachyphrynium braunianum*) are seeds consumed by the Baka.

Hunter-gatherer people are distributed throughout the Congo basin in Africa. They conform several genetically and ethno-linguistically distinct groups [24], broadly subdivided into western groups such as the Baka and Aka, and eastern groups comprising the Efe and Asua. All live mainly in tropical rainforests as forest foragers and hunter-gatherers although two groups, the Bedzan (Medzan) of Cameroon and the Twa of Rwanda and Burundi, inhabit non-forest areas [25]. Although the preeminent traditional way of life for these groups remains associated with forest hunting and gathering, most contemporary groups have taken up some form of agriculture. A typical example are the Baka of the western Congo basin who are distributed in four different countries with the majority living in Cameroon, numbering about 40,000 individuals [26]. Since the 1950s, Baka became sedentarised following missionary activities and the “development assistance” programs by the State after independence [11, 26]; the adoption of agriculture and semi-sedentary lifestyle has been mostly voluntary [27].

Documenting the types of plants used by indigenous people is becoming more urgent as these communities change away from natural diets containing WEP toward domesticated cultigens and processed foods. In this paper, we document WEP use by sedentarised Baka communities in the Mintom region in Cameroon, recording the different usage, and quantifying daily amounts consumed.

## Methods

### Study site

**Fig. 1:**
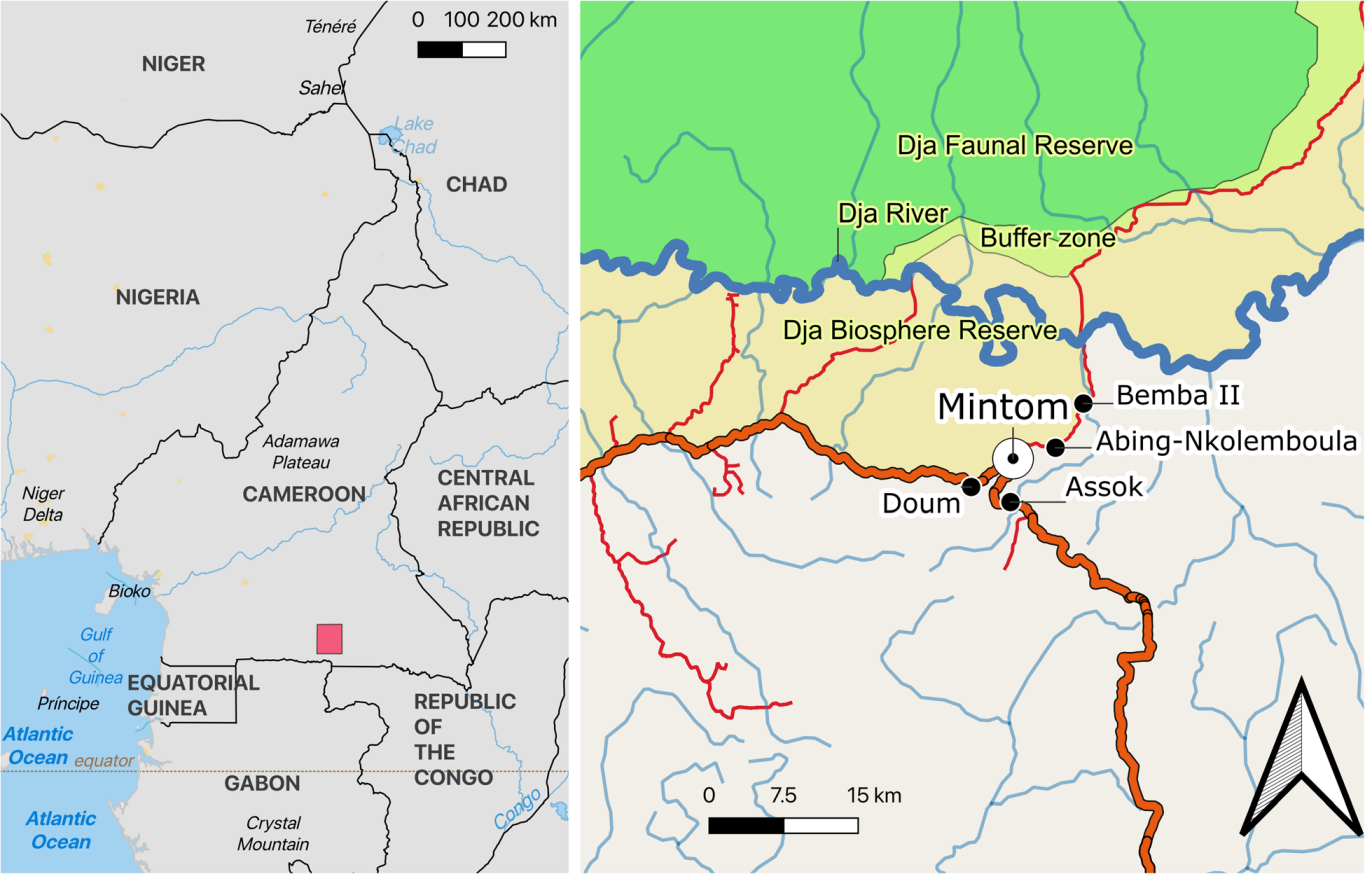
Map of Cameroon showing the study villages surveyed during the present study. Source: public domain map data from Open Street Map, diva-gis (www.diva-gis.org) and Natural Earth (www.naturalearthdata.com)

The study region is located in the eastern part of the Division of Dja et Lobo in south-eastern Cameroon, south of the Dja Faunal Reserve, and the Dja Biosphere Reserve (Fig. 1). Four study villages were selected near the provincial capital Mintom. Mintom has about 6000 inhabitants and is located about 30 km South of the Dja Reserve and 300 km east of the State capital Yaoundé: Assok (15 km East of Mintom), Bemba II and Abing-Nkolemboula (20 and 15 km north, respectively), and Doum (8 km west). Population censuses conducted by us recorded 76 inhabitants in Assok, 62 in Bemba II, 59 in Abing-Nkolemboula, and 109 in Doum during the study period. These villages are predominantly Baka. Interspersed between a total of about 30 Baka villages are about 50 villages exclusively inhabited by major ethnic communities part of the Bantu language group.

Hunter-gatherer groups have witnessed the gradual reduction of access to forest resources [28]. After relocation from the forest, Baka have opened their own plots to grow subsistence crops such as plantain, banana, and cassava [29]. This change in lifestyle has been associated with a marked decline in physical and mental health [30]. Farming has increased in recent years in our study villages, particularly as a result of agricultural programs initiated by our study partner Zerca y Lejos (ZyL) [31, 32], a Spanish NGO working on development and providing health support to Baka communities in the region. Supplementing their life in the village with time in forest camps has led to reduced stress and has helped them maintain better nutritional status [9]. Hunting, fishing, and gathering depend on both the agricultural timetable and season [33].

The climate is equatorial and humid. Rainfall averages between 1500 and 2000 mm per year, and some precipitation is common even during the dry seasons [34]. Mean annual temperature is 25 °C, fluctuating slightly between seasons. There are four seasons: a major dry season is from December to March, a minor rainy season from March to May, a minor dry season in August, and a major rainy season from September to November [10]. The terrain of the region is sloping with gently rolling hills ranging between 250 and 800. The major vegetation type is a mixture of evergreen and semi-deciduous forests [35]. Three broad categories of forests can be distinguished in the Mintom area: forests on rocks, forests on firm soil, and aquatic or hydromorphic forests. Forests on firm soil are divisible into primary and secondary forests.

### Data collection

Ethical approval was not required in this study, although it meets the guidelines of the Social Research Association [36]. Permission to undertake field work in our study area was granted by the Ministry of Scientific Research and Innovation (MINRESI), via the Center for International Forestry Research (CIFOR) in Cameroon. Authorization to work with human subjects was covered by the Arrete No. 00034/A/MINATD/DAP/SDLP granted by the Ministère de l’Administration Territoriale et de la Décentralisation of the Government of Cameroon to ZyL.

In following the principle of free, prior, and informed consent (FPIC), allowing our study communities to give or withhold consent to our project, the Cameroonian field team first organized a meeting with each village in January 2019. All workshops, undertaken in Fang, the lingua franca between the Baka and the local Bantu-speaking farmers, were led by two members of our team, assisted by three local facilitators. The objectives of the project were presented and the interviewers were introduced to the villagers.

Interviews were conducted between January and March 2019, following a pre-prepared open-ended questionnaire. To facilitate communication with the villagers, each interviewer was assisted by a Baka guide from each village, who spoke both French and the Baka language. The guide verbally translated our questionnaire from French to the Baka language (Additional file). Questions were asked to all members of an interviewed household jointly and every answer was noted. General information was first gathered on name, village, ethnic group, age, and sex of the respondents. Questions related to plant (wild and domesticated) use were “to what extent food usage (mode of use) was associated to which plant species” rather than asking “which plants were used for which food usages.” For each mode of use cited (drink, fruit, ingredient, main course, vegetable) we recorded the vernacular Baka name of the plant, plant parts used, the method of harvesting (collecting, cutting, digging), distance from the village for collecting the plant and period of collection during the year. While a “quotation” lists any plant/usage combination by any household irrespective on how often it is cited by different people, “recipes” represent unique species/plant organ/usage combinations. A rarefaction analysis by stepwise addition of informants was conducted to estimate how the addition of informants increased the number of plant species and recipes.

Harvested edible plants, including agricultural plants and WEP, were recorded daily for 14 days between the 22nd of October and the 7th of November 2019 in Assok and Doum. This period encompassed the major rainy season, when mobility into the forest for hunting and gathering is highest among the seasons [10]. Each item destined to be consumed was weighted, and the vernacular names and use were noted.

Plant specimens listed by informants were collected with assistance from the Baka guides. Some plants, mainly trees, were identified in the field but all others were deposited at the National Herbarium of Cameroon (HNC) in Yaoundé. At the HNC, all specimens were first sterilized with alcohol at 90 °C, dried with hot air, and then kept at 20 °C for 3-4 days and sprayed with insecticides. Specimens were identified to the genus level and, whenever possible, to the species level by comparing them with specimens in the herbarium, local field, and identification guides [35, 37, 38], and online databases [39–41].

A total of 73 Baka households provided information on the use of WEP and cultivated plants; 18 households in Assok, 23 in Bemba II, 16 in Doum, and 16 in Abing-Nkolemboula. Information was provided by 21 women (28.8%), 46 men (63%), and 6 couples (8.2%), who were between 18 and 80 years old (average 42 years).

### Data analysis

Diversity indices used are those often employed to assess the diversity in systematic botany or forest ecology [42] such as the Shannon-Weaver index [43], the Simpson index [44], and the regularity or the equitability index of Pielou [45]. The Shannon Weaver index (H’) allows to assess the diversity level of each group taking into account the proportion of each plant in the group. The Simpson index (D) measures the probability for two citations withdrawn randomly from a given group to belong to the same plant or recipe. The regularity or the equitability index of Pielou measures the diversity level reached by a group compared to its maximal level of diversity. It compares two groups which have different number of individuals. An ANOVA was used to compare diversity indices between gender and villages. The amounts of consumed cultivated plants and WEPs was compared with the nonparametric Wilcoxon rank-sum test. Data analysis was performed using R version 3.5.1 [46].

## Results

### Diversity of WEPs

**Fig. 2:**
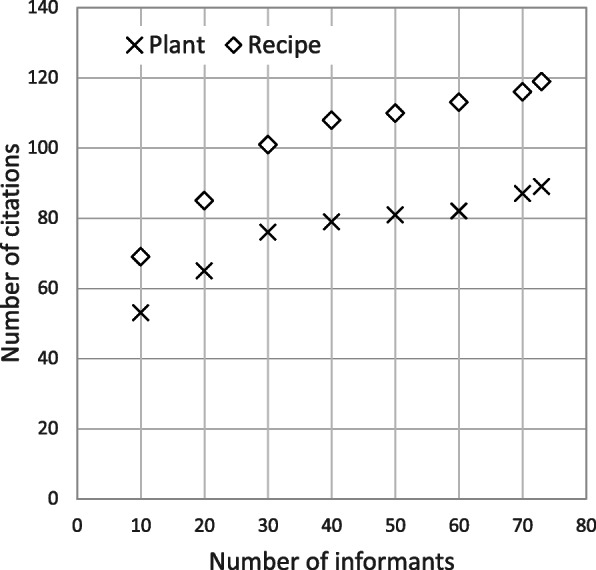
Rarefaction analysis for the number of citations (plants and recipes, i.e., unique combinations of organs of species and their use) dependent of the number of informants. Informants were included in the sequence of the interviews

A total 1519 citations from 88 different plant species were recorded. The plant citations ranged between one and 45 per informant (mean 20.8). We also identified 119 recipes, i.e., unique species/organ/usage combinations. Rarefaction analysis shows that the information collected did not reach a saturation plateau (Fig. 2).

Interviewed women (*n* = 21) reported the use of 69 species and 86 recipes, men (*n* = 46) described 77 species and 100 recipes, and couples (*n* = 6) a total of 51 plants and 59 recipes. Diversity indices (Table 1) indicate an overall high diversity. Average usage densities were 1.2 plants/informant and 1.6 recipes/informant. Values were highest among couples, high for women alone, and low for men alone with a significantly higher citations/informant ratio (ANOVA, df = 2, *F* = 19.06, *p* < 0.001) and Shannon index (ANOVA, df = 2, *F* = 5.9, *p* = 0.003) for citations given by women versus men. The same holds for recipes (ANOVA, df = 2, *F* = 40.55, *p* < 0.001 and df = 2, *F* = 11.47, *p* < 0.001, respectively). Diversity parameters varied largely between villages (Table 2). For the Shannon index, the null hypothesis of the same mean for all villages was rejected for recipes (ANOVA, df = 3, *F* = 5.5, *p* = 0.001) but not for plant diversity (ANOVA, df = 3, *F* = 1.87, *p* = 0.13). With the exception of the Simpson index, the diversity parameters for the two villages Bemba II and Abing-Nkolemboula, which were located on minor gravel roads and were nearer to the Dja Reserve, were in general larger than for the two villages Doum and Assok, which were located on the major tar road and were further away from the Dja Reserve. The values for the Simpson index were the opposite, with the values for Doum and Assok being smaller than for Bemba II and Abing-Nkolemboula.

**Table 1:** Diversity indexes for WEP cited by women, men, and couples

Diversity parameters	Women single		Men single		Women and men		Total	
	Plants	Recipes	Plants	Recipes	Plants	Recipes	Plants	Recipes
Number of informants	21		46		6		73	
Number of citations (Ni)	439		894		186		1519	
Richness	69	86	77	100	51	59	88	119
Density	3.29	4.10	1.67	2.17	8.50	9.83	1.21	1.63
Shanon (H)	5.06	3.75	4.95	3.30	5.44	4.80	4.96	2.99
Pielou (€)	0.86	0.64	0.84	0.56	0.93	0.82	0.84	0.51
Simpson (D)	0.03	0.01	0.04	0.01	0.03	0.02	0.03	0.01

**Table 2:** Diversity indexes for WEP in the four study villages

Diversity parameters	Assok		Doum		Bemba		Nkolemboula	
	Plants	Recipes	Plants	Recipes	Plants	Recipes	Plants	Recipes
Number of informants (Inf)	18		16		23		16	
Number of quotations (Ni)	259		312		505		443	
Richness	38	44	40	49	59	80	54	66
Density	2.11	2.44	2.50	3.06	2.57	3.48	3.38	4.13
Shanon (H)	4.53	4.90	4.66	5.20	5.24	5.83	5.49	5.68
Pielou (€)	0.86	0.90	0.88	0.93	0.89	0.92	0.95	0.94
Simpson (D)	0.06	0.04	0.05	0.03	0.03	0.02	0.03	0.02

### WEP species, parts, and recipes

**Fig. 3:**
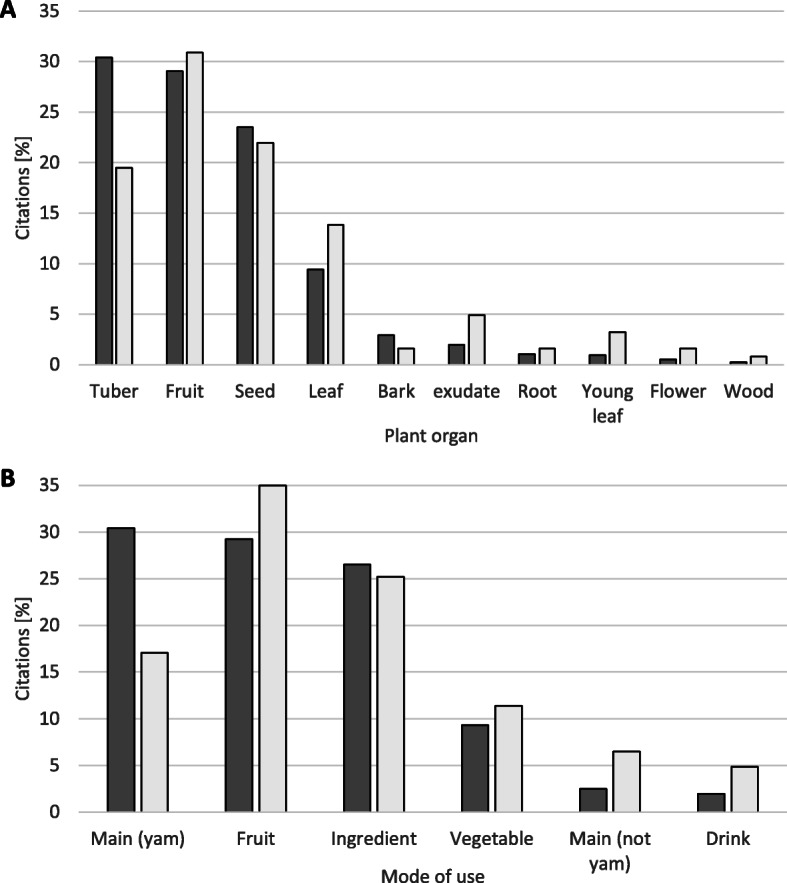
Plant organs (**a**) and usage (**b**). Citations are quotations (dark) and recipes (light)

The 88 plant species belonged to 61 genera and 43 families. Details on species, recipes, and citations are given in Table 3. Eight types of plant organs and exudates from organs of tubers, fruits, and leaves were the most used (Fig. 3a). Six types of use were quoted (Fig. 3b) with yams (*Dioscorea* spp.) being the most consumed, followed by fruits and ingredients. In general, tubers and yams were more often quoted as consumed in the cited recipes.

**Table 3:** List of plant species, voucher number at the National Herbarium of Cameroon (YA)

Vernacular name	Species	Family	Voucher #	Season	Distance	Citations (***n***)	Plant part	Type of usage	Literature
Bitantan	*Abelmoschus esculentus* (L.) Moench	Malvaceae	Bil73	d + r	c	1	Leaf	Vegetable	Cameroon [47]
Pouloue	*Adenia cissampeloides* (Planch. ex Hook.) Harms	Passifloraceae	Bil230	r	c	6	Fruit	Fruit	Congo [22]
Ndiyi na gbeugbeu	*Aframomum daniellii* (Hook. f.) K. Schum.	Zingiberaceae	Bil2	?	f	3	Leaf	Ingredient	
							Fruit	Fruit	Cameroon [20, 21, 48, 49], Congo [22]
Ndiyi na gdi	*Aframomum sulcatum* (Oliv. & Hanb. ex Bak.) K. Schum.	Zingiberaceae	Bet 15	?	c	3	Leaf	Ingredient	
							Fruit	Fruit	Cameroon [20]
Nguimba	*Afrostyrax lepidophyllus* Mildbr.	Huaceae	Nat 70	d + r	Both	108	Bark	Ingredient	Cameroon [20, 21, 50–52], Congo [22]
							Flower	Ingredient	
							Fruit	Ingredient	
							Leaf	Ingredient	
							Root	Ingredient	
							Root	Vegetable	
							Seed	Ingredient	Cameroon [10, 20, 51–53], Congo [22]
							Wood	Ingredient	
Pwa kata	*Agelaea pentagyna* (Lam.) Baill. (syn: *A. obliqua*)	Connaraceae	Bet314	d + r	c	8	Leaf	Vegetable	
Pwa Yando	*Alchornea floribunda* Müll. Arg.	Euphorbiaceae	Bil35	d + r	c	11	Leaf	Vegetable	
Ngongou	*Anonidium mannii* (Oliv.) Engl. & Diels	Annonaceae	Bil115	?	f	16	Fruit	Fruit	Cameroon [20]
Mgbé	*Antrocaryon klaineanum* Pierre	Anacardiaceae	Bil287	r	Both	7	Fruit	Fruit	Cameroon [54], Congo [22]
Mabé	*Baillonella toxisperma* Pierre	Sapotaceae	Bil75	r	Both	112	Fruit	Fruit	Cameroon [20], Congo [22]
							Seed	Ingredient	Cameroon [10, 20, 47, 48, 51, 52], Congo [22]
Fhandako	*Calpocalyx dinklagei* Harms	Mimosaceae	Bil146	d + r	Both	5	Fruit	Fruit	
Alamba na bélé	*Capsicum frutescens* L.	Solanaceae	Bil18	d + r	Both	15	Fruit	Ingredient	Cameroon [47], Congo [22]
Motoubéloubé	*Carapa procera* DC.	Meliaceae	Bil136	r	Both	7	Seed	Fruit	Congo [22]
Monono	*Carpolobia alba* G. Don	Loganiaceae	Bil155	r	c	2	Fruit	Fruit	Cameroon [20, 48], Congo [22]
Ligo	*Cola acuminata* (P. Bwaterv.) Schott & Endl.	Sterculiaceae	Bil161	r	Both	3	Seed	Fruit	Cameroon [20, 21, 48, 49, 53], Congo [22]
Mécor	*Cola rostrata* K. Schum.	Sterculiaceae	Bil137	r	Both	6	Seed	Fruit	Congo [22]
Mengoumé	*Coula edulis* Baill.	Olacaceae	Bil 322	d + r	Both	6	Seed	Fruit	Cameroon [20, 47, 48]
Fawouaboka	*Desbordesia glaucescens* (Engl.) Tiegh.	Combretaceae	Bil130	?	f	2	Seed	Fruit	
Mgbii	*Dicranolepis disticha* Planch.	Thymeleaceae	Bil323	d + r	Both	14	Fruit	Fruit	
							Leaf	Ingredient	
							Tuber	Main	
							Y-leaf	Ingredient	
Kèkè	*Dioscorea burkilliana* Miège	Dioscoreaceae	Bil324	d + r	Both	35	Tuber	Main yam	Cameroon [10, 17, 51], Congo [22]
Esssendé	*Dioscorea hirtiflora* Benth	Dioscoreaceae	Bil325	d + r	f	13	Tuber	Main yam	
Ba’a	*Dioscorea mangenotiana* Miège	Dioscoreaceae	Bil39	d + r	f	56	Tuber	Main yam	Cameroon [17, 51], Congo [22]
Koukou	*Dioscorea munutiflora* Engl.	Dioscoreaceae	Bil295	d + r	Both	42	Tuber	Main yam	Congo [22]
Saba	*Dioscorea praehensilis* Benth	Dioscoreaceae	Bil31	d + r	Both	61	Tuber	Main yam	Cameroon [10, 17, 51], Congo [22]
Essouma	*Dioscorea semperflorens* Uline	Dioscoreaceae	Bil 326	d + r	Both	58	Leaf	Vegetable	
							Tuber	Main yam	Congo [22]
							Y-leaf	Vegetable	
Baloko	*Dioscorea smilacifolia* De Wild.	Dioscoreaceae	Bil94	d + r	f	25	Tuber	Main yam	Cameroon [17], Congo [22]
Ndondo	Dioscorea sp1	Dioscoreaceae	Bil309	d + r	f	32	Tuber	Main yam	
Booli	Dioscorea sp2	Dioscoreaceae	Bil327	d	f	9	Tuber	Main yam	
Boto	Dioscorea sp3	Dioscoreaceae	Bil328	d	f	2	Tuber	Main yam	
Koubé	Dioscorea sp4	Dioscoreaceae	Bil329	r	f	8	Tuber	Main yam	
Djakaka	Dioscorea sp5	Dioscoreaceae	Bil330	d	f	43	Tuber	Main yam	
Efhanguè	Dioscorea sp6	Dioscoreaceae	Bil331	d	f	34	Tuber	Main yam	
Ekorra	Dioscorea sp7	Dioscoreaceae	Bil264	d	Both	1	Tuber	Main yam	
Esopo	Dioscorea sp8	Dioscoreaceae	Bil332	d + r	f	6	Tuber	Main yam	
Fhafhè	Dioscorea sp9	Dioscoreaceae	Bil333	d + r	f	2	Tuber	Main yam	
Mbooto	Dioscorea sp10	Dioscoreaceae	Bil334	d + r	f	2	Tuber	Main yam	
Moussokofandè	Dioscorea sp11	Dioscoreaceae	Bil335	d + r	Both	1	Tuber	Main yam	
Paper	Dioscorea sp12	Dioscoreaceae	Bil336	d	f	2	Tuber	Main yam	
Scèndè	Dioscorea sp13	Dioscoreaceae	Bil337	d	c	1	Tuber	Main yam	
Diya	Dioscorea sp14	Dioscoreaceae	Bil30	d	f	3	Tuber	Main yam	
Bii	*Dioscoresphyllum cumminsii* (Stapf) Diels.	Menispermaceae	Bil338	d + r	f	23	Tuber	Main	Congo [22]
Manjoubou	*Diplazium welwitschii* (Hooker) Diels	Athyriaceae	Bil163	d + r	Both	28	Teaf	Vegetable	Congo [22]
							Y-leaf	Vegetable	
Vin de palme (Gobila)	*Elaeis guineensis* Jacq.	Arecaceae	Bil339	?	?	4	Exu-date	Drink	Congo [22]
Tokomboli	*Eriocoelum macrocarpum* Gilg ex Radlk.	Sapindaceae	Bil271	r	Both	38	Fruit	Main	Congo [22]
							Fruit	Fruit	
							Seed	Main	
							Seed	Fruit	
Bambou	*Gambeya africana* (G. Don. ex Bak.) Pierre	Sapotaceae	Bil256	r	Both	27	Fruit	Fruit	
							Seed	Fruit	Congo [22]
Mpkom	*Garcinia kola* Heckel	Clusiaceae	Bil340	?	f	1	Seed	Fruit	Cameroon [20, 21, 48, 49, 53], Congo [22]
Bemba	*Gilbertiodendron dewevrei* (De Wild.) Léonard	Caesalpiniaceae	Bil341	r	c	1	Fruit	Fruit	Congo [22]
Koko	*Gnetum africanum* Welw.	Gnetaceae	Bil147	d + r	Both	57	Leaf	Vegetable	Cameroon [10, 20, 21, 47, 48, 50, 53–56], Congo [22], Democratic Republic of Congo [57]
Yoloyolo	*Gymnanthemum amygdalinum* (Delile) Sch. Bip. ex Walp.	Asteraceae	Bil29	?	c	1	Leaf	Vegetable	
Essang	*Hibiscus sabdarifa* L	Malvaceae	Bil342	?	c	1	Leaf	Ingredient	
Mingaignai	*Hua gaboni* Pierre ex De Wild.	Huaceae	Bil343	?	f	4	Bark	Ingredient	
							Seed	Ingredient	
Payo	*Irvingia excelsa* Mildbr.	Irvingiaceae	Bil160	d + r	Both	48	Fruit	Fruit	Cameroon [10], Congo [22]
							Seed	Ingredient	
Pféké	*Irvingia gabonensis* (Aub. Lec. Ex O’R.) Baill.	Irvingiaceae	Bil65	r	Both	89	Fruit	Fruit	Cameroon [20], Congo [22]
							Seed	Ingredient	Cameroon [10, 20, 21, 47–54, 56, 58], Congo [22]
Bokoko	*Klainedoxa gabonensis* Pierre	Irvingiaceae	Bil344	?	f	3	Seed	Fruit	Congo [22]
Mapkwa	*Landolphia foretiana* (Pierre ex Jumelle) Pichon	Apocynaceae	Bil141	d + r	oth	13	Fruit	Fruit	Congo [22]
Kwakata	*Lasiodiscus* sp.	Rhamnaceae	Bil345	?	f	12	Leaf	Vegetable	
Ngoka	*Lophira alata* Banks ex Gaertn.	Ochnaceae	Bil346	?	f	1	Fruit	Fruit	
Ngongo	*Megaphrynium macrostachyum* (Benth.) Milne-Redh.	Maranthaceae	Bil347	?	f	1	Fruit	Fruit	Cameroon [21], Congo [22]
Mbée	*Momordica charantia* L	Cucurbitaceae	Bil348	r	c	2	Fruit	Fruit	
Djingo	*Monodora tenuifolia* Benth	Annonaceae	Bil191	?	f	18	Seed	Ingredient	Cameroon [20, 47, 49, 53]
Kombo	*Musanga cecropioides* R. Br.	Moraceae	Bil55	r	Both	8	Exu-date	Drink	
							Fruit	Fruit	Congo [22]
Ngatta	*Myrianthus arboreus* P. Beauv.	Moraceae	Bil49	r	Both	12	Fruit	Fruit	Cameroon [20], Congo [22]
Mossé	*Nauclea diderrichii* (De Wild. & T. Durand) Merr.	Rubiaceae	Bil134	r	Both	8	Fruit	Fruit	Cameroon [20, 48]
Nganako	*Occimum gratissimum* L	Lamiaceae	Bil349	d + r	c	3	Leaf	Ingredient	Cameroon [47, 53]
Koungou	*Pachypodanthium barteri* (Benth.) Hutch. & Dalz.	Annonaceae	Bil350	r	Both	2	Fruit	Fruit	Congo [22]
Kana	*Panda oleosa* Pierre	Pandaceae	Bil149	d + r	Both	40	Seed	Ingredient	Cameroon [10, 20, 48, 51, 52], Congo [22]
Léca-mgbi	*Pentaclethra macrophylla* Benth	Leguminoseae-Caesalpinioidaea	Bil66	r	c	1	Fruit	Fruit	Cameroon [20]
Mbalaka	*Pentadiplandra brazzeana* Bail.	Pentadiplandraceae	Bil80	r	c	14	Seed	Ingredient	Cameroon [20, 51, 52, 58], Congo [22]
Poivre	*Piper guineense* Schum. & Thonn.	Piperaceae	Bil19	d + r	f	9	Fruit	Ingredient	Cameroon [20, 21, 48, 49, 51–55], Congo [22]
Po’o	*Poga oleosa* Pierre	Anisophylleaceae	Bil57	d + r	Both	63	Fruit	Ingredient	Cameroon [20, 54]
							Fruit	Main	
							Fruit	Fruit	
							Seed	Ingredient	
							Seed	Main	
							Seed	Fruit	
Botounga	*Polyalthia suaveolens* Engl. & Diels	Annonaceae	Bil114	d + r	c	1	Leaf	Vegetable	
Ndémbélembé	*Potomorphe umbellata* (L.) Miq. (syn : *Piper umbellatum*)	Piperaceae	Bil154	d + r	c	9	Y-leaf	Vegetable	Cameroon [55]
Péké	*Raphia mombuttorum* Drude	Arecaceae	Bil103	d + r	c	6	Exu-date	Drink	Cameroon [20]
Gobo	*Ricinodendron heudelotii* (Baill.) P. ex Heck.	Euphorbiaceae	Bil69	d + r	Both	19	Fruit	Ingredient	
							Seed	Ingredient	Cameroon [10, 20, 21, 47–53, 56, 58], Congo [22]
Moudongué	*Salacia* sp	Hypocrataceae	Bil351	?	Both	20	Fruit	Fruit	
Libaba	*Santiria trimera* (Oliv.) Aubreville	Burseraceae	Bil121	d + r	Both	14	Fruit	Fruit	Congo [22]
Ekoungou	*Smilax anceps* Wild.	Smilacaceae	Bil352	d	f	5	Tuber	Main	
Kasso	*Tetracarpidium conophorum* (Müll. Arg.) Hutch. Et Dalz.	Euphorbiaceae	Bil 353	?	f	7	Fruit	Fruit	Cameroon [20], Congo [22]
							Seed	Fruit	
Kpwo-ngo	*Tetracera alnifolia* Willd. Subsp. Alnifolia	Dilleniaceae	Bil105	?	f	13	Exu-date	Drink	Congo [22]
Basapa	*Tetracera* sp	Dilleniaceae	Bil354	?	f	4	Exu-date	Drink	
Gwassafhè	*Tetracera* sp2	Dilleniaceae	Bil355	?	f	1	Exu-date	Drink	
Djaga	*Tetrapleura tetraptera* (Schum. & Thonn.) Taub.	Mimosaceae	Bil356	?	f	11	Seed	Ingredient	Cameroon [20, 21, 47–49, 51–53], Congo [22]
Poussa	*Treculia africana* Desc.	Moraceae	Bil297	?	f	1	Seed	Main	Cameroon [20], Congo [22]
Ngoyo	*Trichoscypha acuminata* Engl.	Anacardiaceae	Bil249	r	Both	51	Fruit	Fruit	Cameroon [10, 20], Congo [22]
							Seed	Fruit	
Mongolla	*Trichoscypha arborea* (A. Chev.) A. Chev.	Anacardiaceae	Bil274	r	Both	28	Fruit	Fruit	Cameroon [10, 20], Congo [22]
Séngui1	*Uapaca paludosa* Aubrév. & Léandri	Euphorbiaceae	Bil357	r	Both	14	Fruit	Fruit	
Séngui2	Uapaca guineensis	Euphorbiaceae	Bil50	?	f	5	Fruit	Fruit	
Moundiyè	*Xylopia hypolampra* Mildbr.	Annonaceae	Bil79	?	f	7	Flower	Ingredient	
							Leaf	Ingredient	
							Seed	Ingredient	Cameroon [20, 48, 49, 53]

Collection season (*d* dry season, *r* rainy season, *d + r* all year round, *?* unknown), distance (*n* near, *i.e.* less than 1 km from village, *f* far, both, *?* unknown), number of citations, plant parts, usage, and occurrence in other sites as cited in the literature [10, 17, 20–22, 47–60]

The most widely represented families were Dioscoreacea (21 species/436 citations/23 recipes), Euphorbiaceae (5/56/7), and Anonaceae (5/44/4). The most used WEP in terms of citations and recipes were: *Baillonella toxisperma* (112 citations/2 recipes), *Afrostyrax lepidophyllus* (108/8), *Irvingia gabonensis* (89/2), *Poga oleosa* (63/6), *Dioscorea praehensilis* (61/1), *D. semperflorens* (58/3), *Gnetum africanum* (57/1), *Dioscorea mangenotiana* (56/1), and *Trichoscypha acuminata* (51/2). The most cited recipes included yam tubers (*Dioscorea praehensilis*, *D. mangenotiana*, *D. semperflorens*, *and D. munutiflor*) as main courses, leaves of *Gnetum africanum* as vegetable, seeds of *Baillonella toxisperma*, bush mango *Irvingia gabonensis*, *Afrostyrax lepidophillus*, and *Panda oleosa* as ingredients, as well as the fruits of *Baillonella toxisperma*, *Irvingia gaboneneis*, and *Trichoscypha acuminate*.

### WEP access to the Baka community

A total of 1505 citations included the distance of WEP collection from the village. The WEPs for 76.6% of citations were collected one or more kilometer away from the village compared to 22.5% close to the village. Except for Abing-Nkolemboula with 22.2% of citations, interviewees from the three other villages collected WEP 1 km or more away from the village (Assok: 99.6%, Bemba II: 100%, Doum: 98.1%). Collection distances are shown in Table 3. A total of 17 plants were exclusively collected near villages, 35 plants one or more kilometer away and 35 plants at any distance.

Information on when plants were collected was obtained from 753 separate citations. Tubers were mainly harvested during the dry season (69.4%), and fruits and seeds during the rainy season (82.3%). Barks, exudates, and leaves were harvested during the whole year. A total of 44% of citations were collected during the dry seasons, 39% during the wet season, and 17% throughout the year. The 753 citations with seasonal information involved 66 plants (Table 3), collected during the dry season (14%), wet season (35%), or both (51%).

**Fig. 4:**
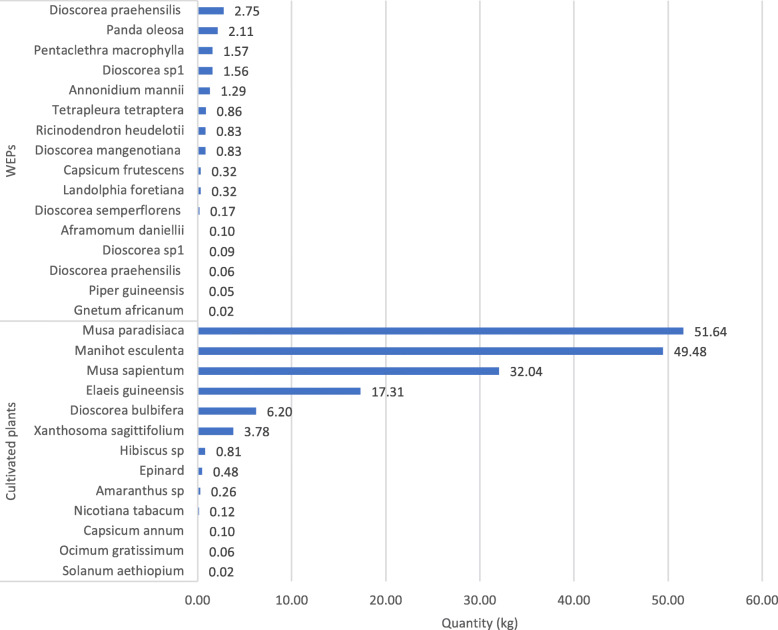
Weight of plants consumed during a 2-week period in the main wet season

We recorded the daily weight of plants consumed in 27 households (Assok: 8, Doum: 19); a total of 99 measurements. As many as 27 different plant items were derived from agricultural plants (*n* = 13) and WEP (*n* = 14). WEP included fruit (1.7 kg), tubers (5.5 kg), seeds (5.4 kg), and leaves (0.02 kg), and cultivated plants included fruit (102.2 kg), tubers (58.5), and leaves (1.7 kg) over the 2-week period. *Musa paradisiaca* (plantain: 51.6 kg), *Manihot esculenta* (cassava: 49.5 kg), *Musa sapientum* (banana: 32.0 kg), and *Elaeis guineensis* (palm oil: 17.3 kg) were the four main cultivated plants consumed, while yams (5.5 kg; 4 species) were the most harvested items among WEP (Fig. 4). The average number of products consumed per day in a household varied significantly between cultivated plants (mean 4.4 ± 4.1 kg, median 3.2 kg, *n* = 37) and WEP (mean 1.0 ± 1.2 kg, median 0.5 kg, *n* = 12) with the difference being significant (Wilcoxon rank-sum test, *W* = 348, *p* = 0.003).

## Discussion

Our results indicate that as many as 88 different plant species—including 14 putative, unidentified wild yam species—were consumed by the 73 interviewed Baka families. A total of 119 recipes included WEP. Despite this relatively large number of items identified in our study, the rarefaction analysis indicates that the number of species recorded is not likely to represent all the WEP diversity used in the study area. This is typical for studies where sampling is not conducted across all seasons, as indicated in an ethnobotanical survey in the Bamenda Highlands in western Cameroon [53]. Although we asked for information on WEP use throughout the year, it is likely that the use of some species elude the memory if they are only rarely consumed in a season other than when the interview was conducted. Except the 14 unidentified wild yam species, 17 WEP species had not been reported in any other ethnobotanical survey for the Baka [10]. A total of 51 plant usages were also unreported before.

The stated number of plants in our study is strikingly higher than the number reported in the grassland with some remaining patches of montane and submontane forests of the Lebialem highlands in southwest Cameroon, where only 26 WEP were documented from 300 respondents from 15 communities [55]. Our number of WEP is also double the number of species in the Bamenda highlands study, which was conducted at the same time of the year as our study but only reported 41 plant species by questioning 121 individuals [53]. Besides ecosystem-specific differences, there are two likely causes for the larger WEP diversity in our study site. First, our site south of Dja Faunal Reserve is in a better conservation state than the Lebialem highlands and the Bamenda highlands where the relatively high human population density has resulted in severe biodiversity degradation [53]. In our study area, there is some indication that WEP is over-exploited near settlements as the inhabitants of three out of four villages needed to travel more than 1 km for collection and harvesting. The distance between the location of harvested common species and the village indicates the scarcity of the resource. Ecosystem intactness might be reflected by the distance of villages to the Dja Faunal Reserve and the development of road infrastructure thus explaining why higher plant and recipe diversities were observed in the two more remote villages closer to the reserve. Second, Baka have inhabited the forested areas for millennia, relying on a hunter-gatherer lifestyle. Their extensive traditional knowledge of WEP is likely reflected in the high number of plants used. In contrast, the inhabitants of the Bamenda highlands are mainly from the non-hunter-gatherer Tikares ethnic group, which settled the area in the eighteenth and nineteenth centuries [53].

Ingram and Schure [48] identified 30 “key” NTFP in Cameroon based on social, cultural, environmental, and economic values. Baka in our study use ten and eight species as WEP from the 17 highest scoring and 13 second-highest scoring NTFP species, respectively, highlighting the biological, cultural, and economic importance of the biodiversity in the region. Highest scoring plants are those that are widely consumed and traded and/or are protected including the moabi (*Baillonella toxisperma*), cola nut (*Cola acuminate*), bitter cola (*Garcinia kola*, *Gnetum africanum*), bush mango (*Irvingia gabonensis*), bush pepper (*Piper guineense*), palm wine (*Raphia mombuttorum*, *Ricinodendron heudelotii*), aidon tree (*Tetrapleura tetraptera* and *Xylopia hypolampra*). The second-highest scoring plants are those that are widely traded or consumed, or have multiple uses or are protected or vulnerable. Used by the Baka are the following: *Aframomum daniellii*, cattlesticks (*Carpolobia alba*), noisette (*Coula edulis*), ironwood (*Lophira alata*, *Megaphrynium macrostachyum*), bilinga (*Nauclea diderrichii*), shea nut (*Poga oleosa*), and *Trichoscypha arborea*. All these edible species have been reported from surveys of Cameroonian markets [20]. Clark and Sunderland [61] list seven NTFP for Central Africa, five of which are WEP; all five were used in our study area: bush mango (*Irvingia gabonensis*), *Gnetum africanum*, *Ricinodendron heudelotii*, cola nut (*Cola acuminate*), and moabi (*Baillonella toxisperma*)*.* The latter plus *Irvingia gabonensis* and *Gnetum africanum* are among the plants most cited for Cameroon [21, 48, 50].

The most species-rich genus was *Dioscorea*, the wild yams, with possibly 20 species. This includes *Dioscorea mangenotiana*, a vigorous annual climber that possesses a long-lived root which can attain as much as 60 kg in weight [15]. WEP are a major part of Baka cultural identity, and wild yams in particular play a specific role in their cosmology. Yams are considered as a link between humans, elephants, and the “jengi” spirit, because these three share this symbolic food [5]. For this reason, wild yams have been considered “Cultural Superfoods” [62], which also relates to the notion of a cultural keystone species [63]. The nutritional importance of wild yams is highlighted by the exploitation through “paracultivation,” whereby growth of wild yams is managed in their natural environment, and over-exploitation is largely avoided [15]. The relatively high number of wild yam species in our study concurs with those assumptions, but is in contrast to the observation by Gallois et al. [10]. Although they report that Baka prefer wild yam when readily available, they seem not to be easily available in their study area, explaining the relative low consumption of wild yams observed there. Similarly, Hirai et al. [51] report only three species (*D. mangenotiana*, *D. burkilliana*, *D. praehensilis*) at the northern periphery of the Boumba-Bek National Park in the East Region of Cameroon. Wild yams store starchy reserves in aerial or underground tubers and are the most important source of carbohydrates for many hunter-gatherers of African forests [15]. Cameroon has the highest yam diversity in Africa with 17 probable species [64], followed by Gabon [65] and Congo Brazzaville [66] with 12 species each, Central African Republic with 11 species [67], and Congo Kinshasa with 9 species [15]. We could only identify six species though there were 14 putative species, which the Baka distinguish with separate names. These presumed yam species remain unidentified and should be a prime target for future work to establish whether there are undescribed species in our study area.

Plant foods other than wild yams are also important sources of macro- and micronutrients and energy for millions of people in the Congo Basin. Enquiries conducted in different regions in Cameroon [21, 49, 53, 59], Côte d’Ivoire [68], and in the Democratic Republic of Congo [57] revealed a high proportion of WEP fruits and seeds. The importance of fruits or seeds is linked to their high nutritive value and also to the production and long-term storage of derived products (oils for example). Edible wild fruits play a key role in the well-being of rural communities in developing countries in Africa and elsewhere, since they replace domestic vegetables during shortage periods (e.g., [69]). The daily consumption of some of these fruits may offer protection against some ailments and oxidative stress [70]. The main fatty acids of *Baillonella toxisperma* oils are oleic, stearic, and palmitic acids. The fact that the biochemical characteristics and fatty acid profile are comparable to common vegetable oils shows that the *B. toxisperma* oil is a potential source of valuable oil which might be used for edible, cosmetic, pharmaceutical and other industrial applications [71]. Etong and Mustapha [72] found that the oil of the bush mango *Irvingia gabonensis* contains six major fatty acids. Oil extracted can be useful both domestically and industrially. Among vegetables, the widely used species of the *Gnetum* genus are rich in proteins, minerals, and amino acids [73]. Among spices, *Afrostyrax lepidophyllus* has antioxidant, anti-inflammatory, and anti-xanthine oxidase activity [74]. The raphiales and palm trees are known by all the people of the Congo Basin as plants producing wines [22]. However, for the majority of species quoted in this study, especially those not listed as “key” NTFP in Cameroon by Ingram and Schure [48], the main nutritional and pharmacological remain undocumented.

While the Baka use of WEP during the whole year is expected to be significant, our 2-week survey of plants used revealed only 14 WEP species were exploited during that time. This relatively small number of WEP is surprising since the quantitative survey was conducted during the major rainy season, when mobility into the forest for hunting and gathering is highest. On the other hand, a low diversity of wild plants was also observed in the diet of Baka in the same region of Cameroon [10] and by other hunter-gatherers in the Congo Basin [75, 76]. For example, Gallois et al. [10] conducted food recalls for the preceding 24 h of 536 individuals and revealed 14 different WEP. Thus, quantitative surveys of WEP use need to be performed during the whole year, as we have done in this study. During our 2-week food quantification, Baka heavily relied on cultivated starchy foods (cassava, plantain) from their own agricultural production. Although there was a large variance between households, the total weight of consumed cultivated plants exceeded the weight of WEP threefold. Plantain, cassava, banana, and oil palm are the four main cultivated plants used, while wild yams (4 species) represent the most harvested products among the WEP. A similar bias toward cultivated food was observed by Gallois et al. [10]. In their study, starchy foods were cited in 93% of dietary recalls.

Among the BaYaka Pygmies from Congo, knowledge of WEP is widely shared among people regardless of relatedness, while knowledge of medicinal plants is mainly kept between spouses and relatives [13]. It is, therefore, surprising to find such strong sex-specific differences in the information given by men and women in our study site. About twice as many men reported WEP details than women. We noted that Baka women talk scarcely when they are accompanied by their husbands. But when they have an opportunity to be alone Baka women were much more open and provided more information than men on the same subject (high diversity of usages), which explains the higher information densities of plants and recipes, the higher values of the Shannon and Pielou indexes for plants and recipes and the weak values of the Simpson index for plants. All these findings highlight the importance of gathering information from all members of a given family during ethnobotanical surveys.

## Conclusions

Surveys carried out among Baka people living south of the Dja Biosphere Reserve revealed 88 edible plants species including 14 putative but not identified wild yam species (genus *Dioscorea*). This genus was with six identified and 14 putative species the most species-rich genus in the study, emphasizing their nutritional and cultural importance for Baka. Compared to the Bamenda Highlands in western Cameroon, the Baka WEP diversity was more than double. Excluding the 14 unidentified wild yam species 17 WEP species have not been reported in any other ethnobotanical survey including on Baka [10]. The importance of the study area for WEP diversity is also highlighted by the fact that 18 out of the 30 “key” NTFP in Cameroon [48] were quoted by Baka. The increasing influence of market economies on the lifestyle of hunter-gatherers since sedentarization from the 1950s onwards is exemplified by the high proportion of starchy food in daily nutritional intake observed here and elsewhere [10]. Baka still harvest and use a wide variety of WEP, giving the opportunity to further document Baka’s knowledge of WEP especially as biological resources and indigenous knowledge are diminishing with high destruction and a growing disinterest among the younger generation [53]. Fostering this knowledge will be important for sustainable development and achieving food security.

## Supplementary information

**Additional file 1.** : Questionnaire pour les produits forestiers non ligneux (PFNL) utilises dans l’arrondissement de mintom: les plantes alimentaires.

## Data Availability

Plant specimens were deposited in the National Herbarium of Cameroon, Yaoundé.
