# Supplementary material for: Ethnobotanical survey of wild edible plants used by Baka people in southeastern Cameroon
Source: J Ethnobiol Ethnomed. 2020 Oct 22;16:64. doi: 10.1186/s13002-020-00413-0 (PMC7579891; doi:10.1186/s13002-020-00413-0)
Supplement: Supplementary file 1 — Additional file 1. : Questionnaire pour les produits forestiers non ligneux (PFNL) utilises dans l’arrondissement de mintom: les plantes alimentaires. [file 13002_2020_413_MOESM1_ESM.docx]

# Supplementary Appendix 1. Questionnaire

**QUESTIONNAIRE POUR LES PRODUITS FORESTIERS NON LIGNEUX (PFNL) UTILISES DANS L’ARRONDISSEMENT DE MINTOM: LES PLANTES ALIMENTAIRES**

Dans cette enquête, l’unité d’échantillonnage est le ménage. On vise tout le monde, et on part des usages vers les plantes.

Date : …………………………… Nom du village : ………………………………………………..

1. ***Informations générales sur l’enquêté***

Noms et prénoms : …………………………………………………………………………...

Age : …………………………….. ; Sexe : …………………

Ethnie : ……………………………………… ; Profession : ………………………

1. ***Usages des plantes alimentaires***

- Les **plantes de bouche**ou **amuse-gueule**: plantes qui se consomment soit directement, soit aprèstransformation en dehors du repas principal ;
- Les **légumes** : plantes potagères dont les organes (feuilles, fruits, graines, …) sont utilisés pour la confection des plats d’accompagnement
- Le **plat principal**: constitue l’aliment de base du repas des ménages de la zone indiquée ;
- L’**épice** ou **condiment** est une substance aromatique qui sert à assaisonner les mets.
- Pour le **thé**, l’organe végétal est utilisé en décoction.

1. Quel est le met le plus prisé dans le village ?

………………………………………………………………………………

Il est composé de quoi ?

Plante 1 :…………………………………………………………………………..

Organe :……………………………………………………………………………

Plante 2 : ……………………………………………………………………..

Organe : ………………………………………………………………

Méthode préparation : ………………………………………………………………

1. Quelles sont les plantes sauvages que vous consommez comme amuse-gueule ?
   1. Plante 1 :………………………………Organe végétal………………..
   2. Plante 2:…………………………………Organe végétal………………
   3. Plante 3…………………………….. …...Organe végétal
   4. Etc…….
2. Quelles sont les plantes sauvages que vous consommez comme condiment ?
   1. Plante 1 :………………………………………………………………………
      1. Organe végétal……………………………………………………….
      2. Mode de préparation…………………………………………..……..
      3. Observations…………………………………………………………..
   2. Plante 2 :………………………………
      1. Organe végétal…………………………………………………………
      2. Mode de préparation…………………………………………………..
      3. Observations…………………………………………………………..
   3. Plante 3 :………………………………
      1. Organe végétal……………………………………………………….
      2. Mode de préparation………………………………………………….
      3. Observations………………………………………………………….
3. Quelles sont les plantes sauvages que vous consommez comme thé ?
   1. Plante 1………………………..Organe végétal……………………………….
   2. Plante 2………………………..Organe végétal………………………………..
   3. Plante 3…………………………Organe végétal……………………………
